# Diagnostic and prognostic value of angiography-derived index of microvascular resistance: a systematic review and meta-analysis

**DOI:** 10.3389/fcvm.2024.1360648

**Published:** 2024-04-15

**Authors:** Dayang Wang, Xiaoming Li, Wei Feng, Hufang Zhou, Wenhua Peng, Xian Wang

**Affiliations:** ^1^ Cardiovascular Institute, Dongzhimen Hospital, Beijing University of Chinese Medicine, Beijing, China; ^2^ Second Department of Cardiology, Dongzhimen Hospital, Beijing University of Chinese Medicine, Beijing, China; ^3^ Center of Intervention, Dongzhimen Hospital, Beijing University of Chinese Medicine, Beijing, China

**Keywords:** coronary microvascular dysfunction, angiography-derived index of microvascular resistance, diagnostic test, prognostic value, major cardiovascular events, meta-analysis

## Abstract

**Background:**

The angiography-derived index of microvascular resistance (A-IMR) is a novel tool for diagnosing coronary microvascular dysfunction (CMD) addressing limitation of unavailability. However, the clinical value of A-IMR remains controversial.

**Methods:**

A systematic review and meta-analysis was conducted. PubMed, EMBASE, Cochrane Library and Web of Science were searched for relevant studies. Studies that reported estimates of A-IMR's diagnostic accuracy (with thermodilution-based IMR as the reference test) and/or predictions of adverse cardiovascular events were selected. Pooled sensitivity, specificity, area under the summary receiver operating characteristic curve (sROC) were calculated to measure diagnostic performance; pooled hazard/risk ratio (HR/RR) and 95% confidence interval (95% CI) of major adverse cardiovascular events (MACE) or other independent adverse events were calculated to measure prognostic effect. This study was registered with PROSPERO (CRD42023451884).

**Results:**

A total of 12 diagnostic studies pooling 1,642 vessels and 12 prognostic studies pooling 2,790 individuals were included. A-IMR yielded an area under sROC of 0.93 (95% CI: 0.91, 0.95), a pooled sensitivity of 0.85 (95% CI: 0.79, 0.89) and a pooled specificity of 0.89 (95% CI: 0.83, 0.93) for the diagnosis of CMD. CMD diagnosed using A-IMR was associated with higher risks of MACE (HR, 2.73, 95% CI: 2.16, 3.45), CV death (RR, 2.39, 95% CI: 1.49, 3.82) and heart failure hospitalization (HR, 2.30, 95% CI: 1.53, 3.45).

**Conclusion:**

A-IMR demonstrated high diagnostic accuracy for CMD and showed a strong prognostic capability in predicting the risk of adverse CV outcomes.

**Systematic Review Registration:**

https://www.crd.york.ac.uk/prospero/display_record.php?ID=CRD42023451884, PROSPERO (CRD42023451884).

## Graphical Abstract

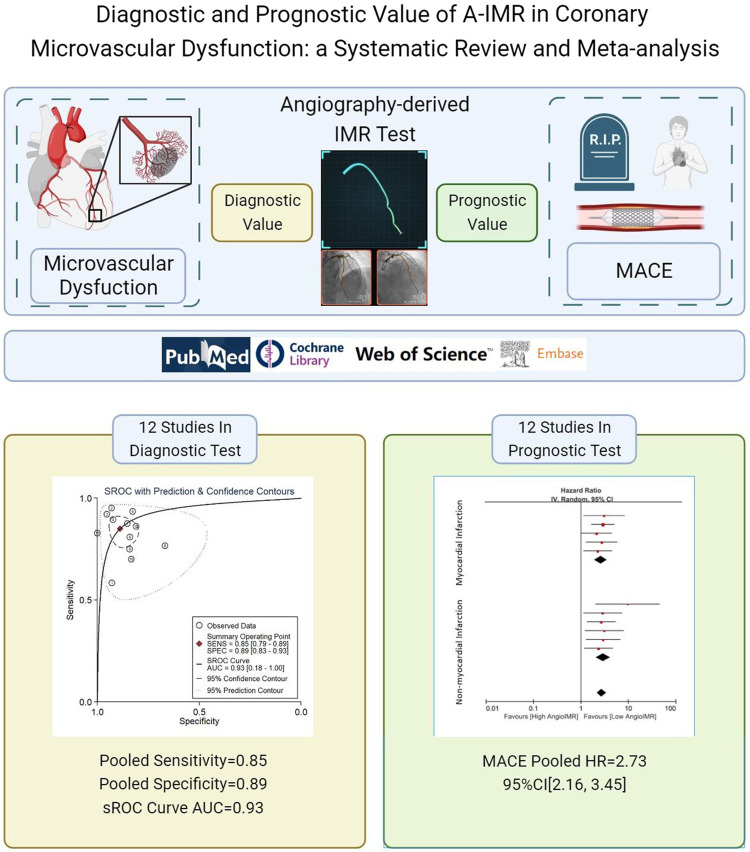

## 1 Introduction

Coronary microvascular dysfunction (CMD) is commonly observed in clinical settings. It relates to abnormalities in the heart's microcirculation, specifically impacting the arterioles and capillaries responsible for regulating myocardial blood flow (1). CMD may arise subsequent to acute myocardial infarction (AMI) (2) and is also evident in patients diagnosed with either obstructive or non-obstructive coronary artery disease (3). Pathogenetically, CMD primarily arises from endothelial dysfunction. However, in the context of AMI, intracoronary thrombosis can lead to microcirculatory embolism, constituting another significant mechanism of CMD. Regardless of its association with AMI, CMD is consistently associated with a poor prognosis (4, 5). Therefore, the identification of CMD is crucial for improving patient outcomes (6).

Currently, the diagnostic approaches for CMD are categorized into invasive and non-invasive strategies. The thermodilution-based index of microvascular resistance (T-IMR) is recommended as the gold standard for the invasive methods (7). However, T-IMR requires adenosine-induced hyperemia, which often introduces chest discomfort and dyspnea (8); the use of additional intra-vascular devices (pressure wire) increases the risk of coronary artery dissection; administering additional heparin to prevent thrombosis can also heighten the risk of hemorrhage. These limitations have restricted the widespread use of the T-IMR test.

For non-invasive strategies, cardiac Positron Emission Tomography (PET) and cardiac magnetic resonance imaging (CMR) are recommended (9). Cardiac PET is currently recognized as the gold standard reference of non-invasive assessment (2); CMR provides high diagnostic accuracy, complemented by their advantages in high-resolution and localization (10, 11). However, both techniques have inherent limitations, including high costs, extended test durations, and constraints associated with venue, equipment, and resource availability (2).

The angiography-based index of microvascular resistance (A-IMR) is a novel approach to CMD diagnosis. A-IMR is based on a principle akin to that of T-IMR. The distinction lies in that, in A-IMR, the blood flow velocity is calculated based on coronary angiography images, while the intracoronary pressure is calculated according to aortic pressure (12). Several A-IMR systems have emerged in recent years, such as QAngio (13), FlashAngio (12), AccuIMR (14) and AngioPlus (15). At present, only limited small-sample clinical studies on A-IMR diagnostic accuracy have been published. Furthermore, the role of A-IMR in predicting clinical outcomes remains unknown.

Therefore, we conducted a systematic review and meta-analysis to evaluate current evidence of A-IMR's diagnostic accuracy for CMD and its associated prognostic significance for adverse cardiovascular (CV) outcomes.

## 2 Methods

### 2.1 Registration

The protocol of this studies was registered with PROSPERO (No. CRD42023451884) (16). This review was conducted and reported according to the Preferred Reporting Items for a Systematic Review and Meta-analysis of Diagnostic Test Accuracy Studies (PRISMA-DTA) (17) and the PRISMA 2020 update (18).

### 2.2 Data sources and searches

The literature searches were conducted in the following four databases: PubMed, EMBASE, Cochrane Library and Web of Science. The publication time was set from the inception to 5th August 2023. We used the following subject terms and free words to perform search: “index of microvascular resistance”, “coronary microvascular dysfunction”, “angiography-derived”, “pressure wire free”, “non-invasive” etc. See Supplementary Table S1 for search strategies in detail.

### 2.3 Eligibility criteria, study selection and data extraction

Studies were considered eligible for inclusion if they met following criterion: (1) were published as a full-length article; (2) were written in English; (3) were cohort study or diagnostic test study; (4) included patients underwent coronary angiography due to confirmed or suspected CAD; (5) reported at least one of the following outcomes: estimates of the diagnostic accuracy of A-IMR compared with invasive T-IMR as the reference test; adjusted hazard ratio (HR) of major adverse cardiovascular events (MACE) with 95% confidence interval (CI) among participants with either a positive or negative A-IMR diagnosed CMD; raw data or risk ratio (RR) for heart failure (HF) hospitalization, CV death and other adverse events. Studies were eligible regardless of whether they included patients with myocardial infarction and regardless of the type/band of A-IMR software/system used.

Two independent reviewers (DW and XL) scanned titles and abstracts according to the inclusion criteria, reviewed full-text articles, and determined their eligibility. Any discrepancy regarding searches and selection was discussed in consultation with and resolved by a third reviewer (WP). The full text was retrieved for further inspection if a study potentially met the inclusion criteria.

Two reviewers independently conducted data extraction. The data included: (1) study-level general information; (2) baseline characteristic of population; (3) outcomes from original eligible sources (the ascertainment of clinical events was accepted as reported). Discrepancy, if any, was verified and resolved by a third reviewer (WP). Records of studies was managed with the Endnote X9 software.

### 2.4 Quality assessment

In the diagnostic test section, the risk of bias and applicability of each study were evaluated using the Quality Assessment of Diagnostic Accuracy Studies, version 2 tool (QUADAS-2) (19). The risk of bias in cohort studies was assessed using the Newcastle–Ottawa Scale (NOS) (20). Specifically, we stipulated that a follow-up duration of at least 1 year would be scored to account for the occurrence of MACE or other events.

### 2.5 Statistical analysis

In the diagnostic test, the presence of a threshold effect was determined by calculating the Spearman correlation coefficient using MetaDiSc 1.4 software (21). If there's no threshold effect, a pooled analysis was conducted.

A bivariate random-effects model was employed for the meta-analysis of diagnostic studies. For each study, raw data of true-positives, true-negatives, false-positives, and false negatives were either extracted from the study or generated from reported diagnostic estimates. Subsequently, sensitivity and specificity were calculated. Study-level findings were summarized using a summary receiver operating characteristic (ROC) curve plot. From the meta-analysis, pooled estimates of sensitivity, specificity, negative likelihood ratio (-LR), and positive likelihood ratio (+LR) for A-IMR were used to generate forest plots. The *I*^2^ statistic was employed to assess inter-study heterogeneity. To further explore the sources of heterogeneity, a meta-regression test was conducted. STATA 14 (22) (StataCorp) with MIDAS module (23) were employed to conduct statistical analyses in diagnostic meta-analysis.

In the prognostic meta-analysis, summary effect size for MACE were calculated using pooled HR. Independent events of all-cause death, CV death, non-fatal AMI, revascularization, HF hospitalization and angina hospitalization were calculated using RR. A random-effects model was used in all the outcomes. We did not conduct pooled analyses for outcomes reported in fewer than three studies. Inter-study heterogeneity was assessed using the *I*^2^ statistic, which was defined as *I*^2^-values of 50% or greater. The *z* statistic was computed for each outcome of interest, and the results were considered statistically significant at 1-sided *p* < 0.05. Meta-analysis results were presented using forest plots.

Subgroup analyses were performed to investigate possible sources of heterogeneity and to assess the effect of specific variables on results, including population of STEMI (after PCI), population of ischemia with non-obstructive coronary arteries (INOCA), prospective cohort, A-IMR systems, and adenosine-induced hyperemia during procedure. To evaluate the robustness, we performed a sensitivity analysis for each outcome. This involved sequentially excluding individual studies to ascertain their impact on the total results. RevMan 5.4.1 software (24) were employed to conduct statistical analyses in the prognostic meta-analysis.

The publication bias was assessed using funnel plots by displaying individual study effect for the outcomes of interest. Funnel plot asymmetry was also evaluated using Deeks' test for diagnostic studies and Egger's test for prognostic studies (with 1-sided *p* < 0.1 indicating significant publication bias). The publication bias assessments were conducted using STATA 14 (22) software.

## 3 Results

### 3.1 Study selection and characteristics

**Figure 1:**
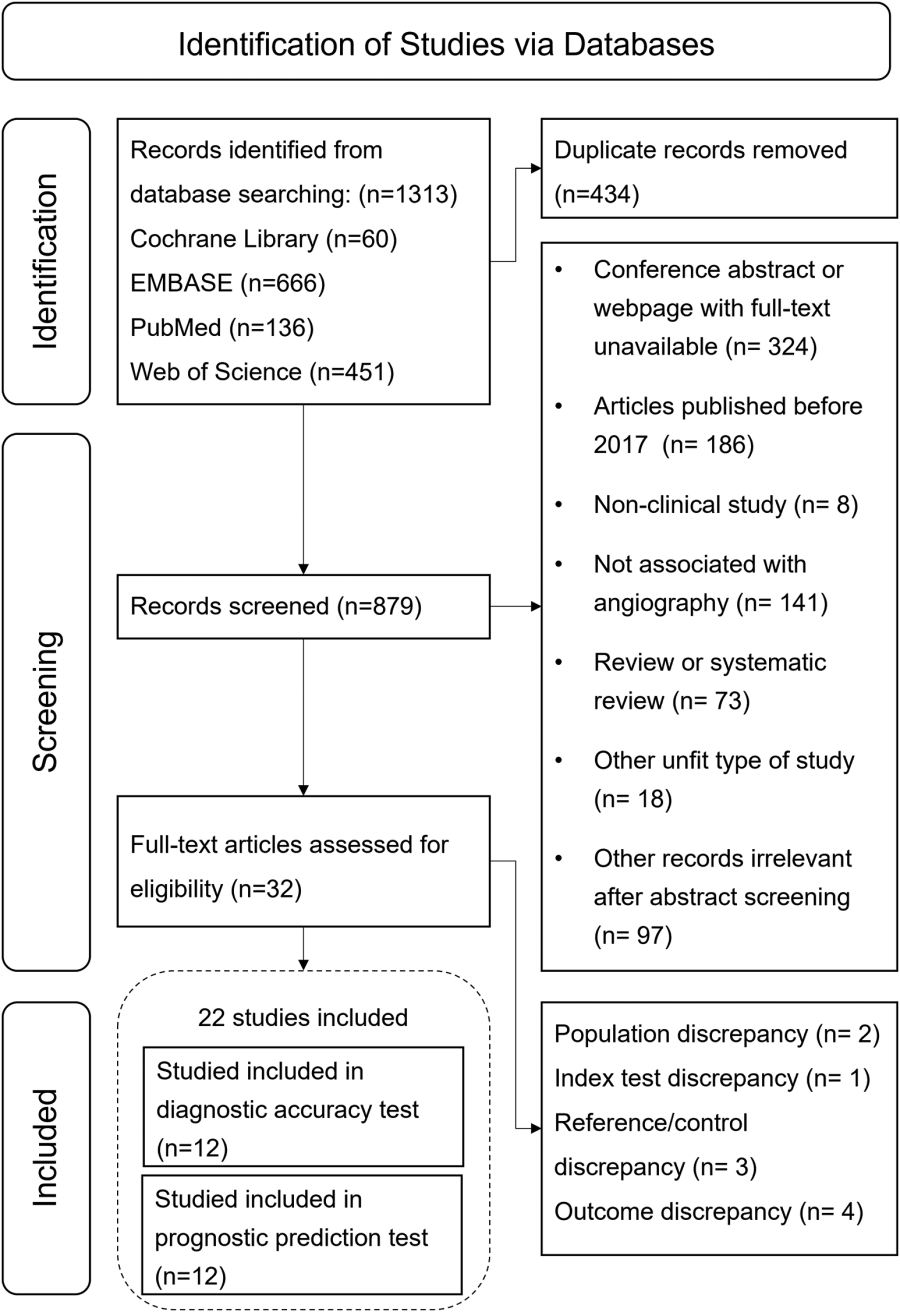
Flow chart of study selection process.

From the initial search, 1,313 records were obtained, of which 434 duplicates were removed. Of the 879 remaining records, 847 were excluded after screening titles and abstracts due to irrelevance with A-IMR or inappropriate article types. After assessing the full text of the remaining 32 articles, we excluded 3 records due to population discrepancies, 2 for reference test discrepancies, 1 for exposure discrepancies, and 4 for outcomes discrepancies. Ultimately, 22 studies met the inclusion criterion and were included in the systematic review. Among them, twelve studies (12–15, 25–32) were included in the meta-analysis of diagnostic test, and twelve in prognostic test (7, 30, 31, 33–41). The study selection process is illustrated in Figure 1.

In the diagnostic meta-analysis, a total of 1,642 lesions were included. Twelve studies encompassed four types of software, including FlashAngio, QAngio, AngioPlus, and AccuIMR. Three studies induced hyperemia during the measurement of A-IMR values. In studies or cohorts involving STEMI, a cutoff value of 40 mmHg·s/mm was used for CMD. In studies without the STEMI population, a 25 mmHg·s/mm cutoff was utilized, with one exception (26). The general information of included studies was displayed in Table 1.

**Table 1:** General characteristic of included studies.

Study ID	Country	Software	Population	Number of lesions/patients^a^	A-IMR cutoff (mmHg·s/mm)	NH A-IMR	Definition of primary outcomes^b^	Follow-up period (year)	Study design
Diagnostic accuracy test^c^									
Ai et al. (12)	China	FlashAngio	INOCA	57	25	Yes	–	–	Prospective
De Maria et al. (13)	U.K.	QAngio	STEMI	92	40	No	–	–	Prospective
Tebaldi et al. (26)	Italy	QAngio	CCS	44	44.2	Yes	–	–	Prospective
Choi et al. (30)	South Korea	FlashAngio	STEMI	31	40	No	–	–	Prospective
Kotronias et al. (31)	U.K.	QAngio	STEMI	262	43	Yes	–	–	Retrospective
Mejia-Renteria et al. (32)	Spain/South Korea	QAngio	CCS	115	25	Yes	–	–	Prospective
Scarsini et al. (20)	U.K.	QAngio							
CCS cohort			CCS	131	25	Yes	–	–	Prospective
NSTE-ACS cohort			NSTE-ACS	63	25	Yes	–	–	Prospective
STEMI cohort			STEMI	52	40	Yes	–	–	Prospective
Fan et al. (15)	China	AngioPlus	CAD	257	25	Yes	–	–	Prospective
Jiang et al. (14)	China	AccuIMR	CCS	239	25	Yes	–	–	Retrospective
Fan et al. (29)	China	AccuIMR							
CCS cohort			CCS	90	25	Yes	–	–	Retrospective
NSTEMI cohort			NSTEMI	85	25	Yes	–	–	Retrospective
STEMI cohort			STEMI	57	40	Yes	–	–	Retrospective
Huang et al. (28)	China	FlashAngio	Suspected INOCA	113	25	Yes	–	–	Prospective
Mejia-Renteria et al. (32)	Spain	QAngio	INOCA	104	25	Yes	–	–	Prospective
Prognostic prediction test									
Abdu et al. (34)	China	FlashAngio	MINOCA	109^a^	43	Yes	①②③⑥⑦	2.0	Retrospective
Choi et al. (30)	South Korea	FlashAngio	STEMI	309^a^	40	No	①②	7.3	Retrospective
Dai et al. (7)	China	FlashAngio	CAD	187^a^	25.1	Yes	①②	2.3	Retrospective
Kotronias et al. (31)	U.K.	QAngio	STEMI	242^a^	43	Yes	②⑤⑧	4.2	Retrospective
Duan et al. (36)	China	FlashAngio	STEMI	213^a^	40	Yes	①②③④⑤⑦	1.0	Retrospective
Feng et al. (39)	China	FlashAngio	CCS	282^a^	25	Yes	①②③④	2.9	Retrospective
Zhang et al. (38)	China	FlashAngio	CCS	290^a^	25	Yes	①②③④	2.9	Retrospective
Liu et al. (35)	China	FlashAngio	INOCA	151^a^	25	Yes	①②③④⑦⑥	2.9	Retrospective
Luo et al. (40)	China	AngioPlus	STEMI	942^a^	25	Yes	②	0.1	Retrospective
Mohammed et al. (37)	China	FlashAngio	HFpEF	137^a^	25	Yes	①②	1.25	Retrospective
Wang B et al. (41)	China	FlashAngio	Post-RA	118^a^	25	Yes	③④⑤	1.8	Retrospective
Wang H et al. (33)	China	AngioPlus	STEMI	506^a^	25	Yes	②③④⑤	1.0	Retrospective

^a^ Number of patients for prognostic cohort.

^b^ Definition of primary outcomes: ①cardiac death; ②readmission of heart failure; ③myocardial infarction; ④revascularization; ⑤all-cause death; ⑥stroke; ⑦angina rehospitalization; ⑧resuscitated cardiac arrest. ^c^In the diagnostic meta-analysis, all studies employed T-IMR as diagnostic references.

IMR, index of microvascular resistance; NH, non-hyperemic; MACE, major adverse cardiovascular events; INOCA, ischemia with non-obstructive coronary arteries; MINOCA, myocardial infarction with non-obstructive coronary arteries; STEMI, ST elevated myocardial infarction; CCS, chronic coronary syndrome; CAD, coronary artery disease; ACS, acute coronary syndrome; HFpEF, heart failure with preserved ejection fraction; PCI, percutaneous coronary intervention; RA, rotational atherectomy.

The prognostic meta-analysis assessed a total of 2,790 patients. Except for one study (40) with a one-month follow-up, the follow-up periods in the remaining 11 studies ranged from 1 to 7.3 years. Primary endpoints of the included studies varied and are detailed in Table 1. Eleven studies employed multivariable analysis to adjust HR. Supplementary Table S2 lists the adjustment of confounders in the included studies. Table 2 displays the baseline characteristics of the 22 studies.

**Table 2:** Baseline characteristic of included studies.

Study ID	Sex (male%)	Age, (year)	Population	A-FFR	HT (%)	DM (%)	Hyperlipidemia (%)	Current smoking	LVEF (%)
Diagnostic accuracy test									
Ai et al. (12)	53.6	61.9	INOCA	–	53.6	50.0	66.1	28.6	65.9
De Maria et al. (13)	77.8	61.5	STEMI	–	62.2	17.7	42.2	57.0	49.3
Tebaldi et al. (26)	77.3	70	CCS	–	77	59	59	-	–
Choi et al. (30)	87.1	63.9	STEMI	–	61.3	38.7	61.3	29.0	56.7
Kotronias et al. (31)	82.1	62	STEMI	–	46	41	39	14	50
Mejia-Renteria et al. (27)	76	64.2	CCS	–	63.5	35.6	43.3	28.8	–
Scarsini et al. (25)				–					
CCS cohort	66.6	67.0	CCS	–	66.6	16.7	47.2	38.9	–
NSTE-ACS cohort	60.5	63.0	NSTE-ACS	–	60.5	13.9	32.6	46.5	–
STEMI cohort	84.8	63.5	STEMI	–	50.0	13.6	36.4	51.5	–
Fan et al. (15)	64.4	62.8	CAD	–	65.6	25.2	35.6	35.6	–
Jiang et al. (14)	69.0	64	CCS	–	59.1	29.1	9.8	21.2	–
Fan et al. (29)	58	64	CAD	–	59	26	32	33	59
Huang et al. (28)	56.6	62.9	INOCA	–	66.1	43.1	25.7	33.9	65.8
Mejia-Renteria et al. (32)	31	61.2	INOCA	–	52	14	42	12	58
Prognostic prediction test									
Abdu et al. (34)	51.4	63.8	MINOCA	0.95 ± 0.02	50.4	17.4	18.4	48.9	54.9
Choi et al. (30)	74.8	61.4	STEMI	0.88 ± 0.09	47.2	50.2	34.3	26.9	51.7
Dai et al. (7)	69.6	65	CAD	0.91 ± 0.06	72.5	36.2	8.0	26.1	59.7
Kotronias et al. (31)	82.1	62	STEMI	0.94 (0.90, 0.98)	46	41	39	14	50
Duan et al. (36)	84.0	58.3	STEMI Post-PCI	NM	49.47	17.84	–	49.29	52.1
Feng et al. (39)	67.7	64.9	CCS	0.92 (0.91, 0.95)	70.75	34.0	20.92	24.11	63
Zhang et al. (38)	69.3	64.8	CCS	0.92 ± 0.06	70	35.1	20.7	24.5	62.3
Liu et al. (35)	41.1	60.6	INOCA	0.93 ± 0.03	50.3	14.6	10.6	15.2	63
Luo et al. (40)	84.6	57.8	STEMI Post-PCI	NM	55.8	16.8	15.1	–	54.9
Mohammed et al. (37)	67.2	72.4	HFpEF	0.9 ± 0.02	71.5	33.6	48.9	21.2	60
Wang B et al. (41)	68.9	72.4	Post-RA	0.92 ± 0.03	78.8	49.2	11.0	25.4	58.0
Wang H et al. (33)	82.2	63	STEMI Post-PCI	NM	52.4	27.1	27.3	45.3	54

A-FFR, angiography derived fractional flow reverse; HT, hypertension; DM, diabetes mellitus; LVEF, left ventricular ejection fraction; INOCA, ischemia with non-obstructive coronary arteries; STEMI, ST elevated myocardial infarction; CCS, chronic coronary syndrome; CAD, coronary artery disease; ACS, acute coronary syndrome; HFpEF, heart failure with preserved ejection fraction; PCI, percutaneous coronary intervention; RA, rotational atherectomy; NM, not mentioned.

### 3.2 Diagnostic meta-analysis

#### 3.2.1 Quality assessment

**Figure 2:**
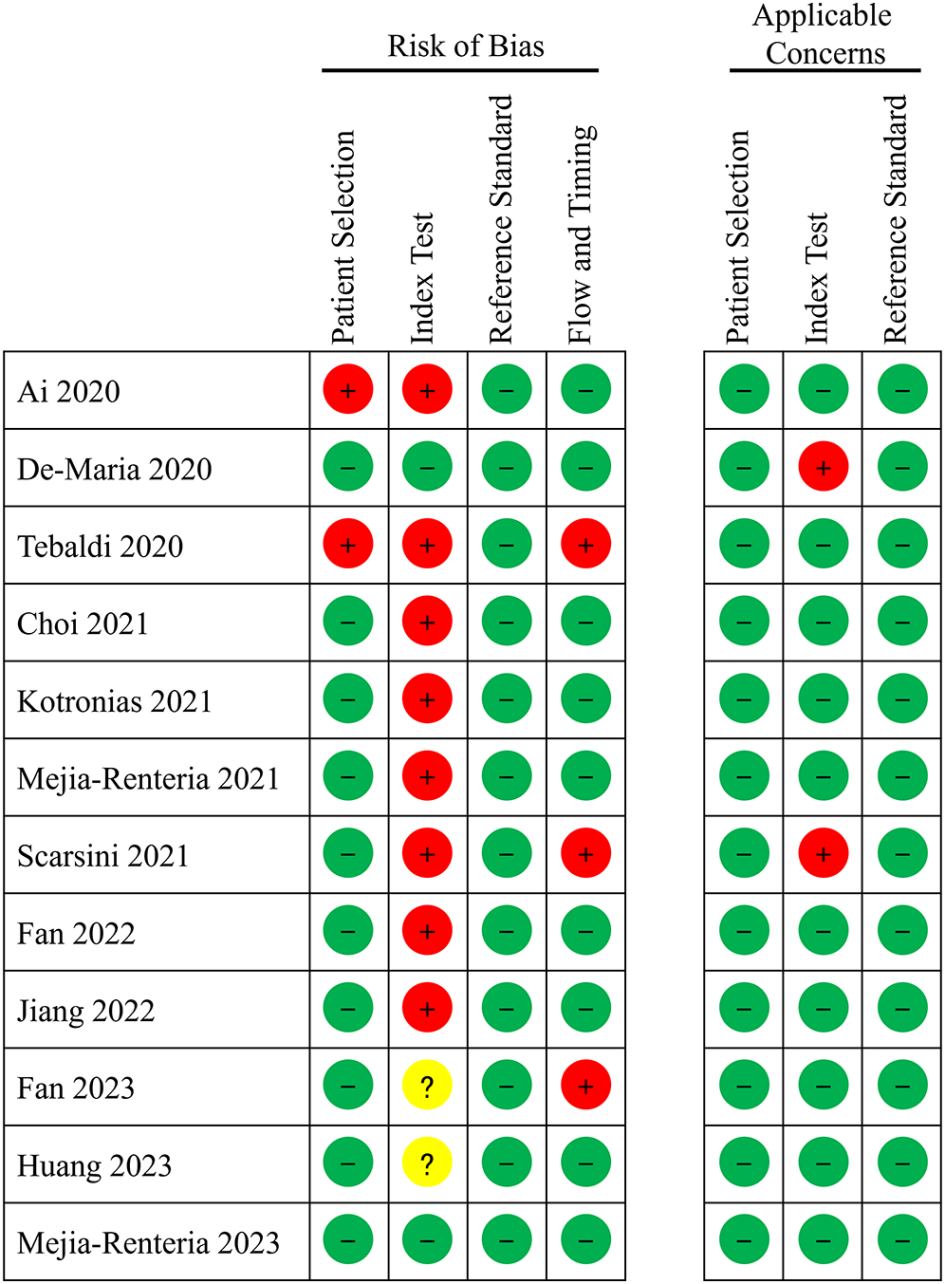
Quality assessment of diagnostic accuracy studies.

The quality of the included studies, as assessed by QUADAS-2, is illustrated in Figure 2. Given that A-IMR is a novel technology, many studies did not prespecify the cutoff value; instead, they determined the optimal cutoff value based on their study data (even though most of these studies converged on a consistent optimal cutoff). Consequently, most of the studies categorized the Index Test domain as “high risk”.

#### 3.2.2 Diagnostic performance

**Figure 3:**
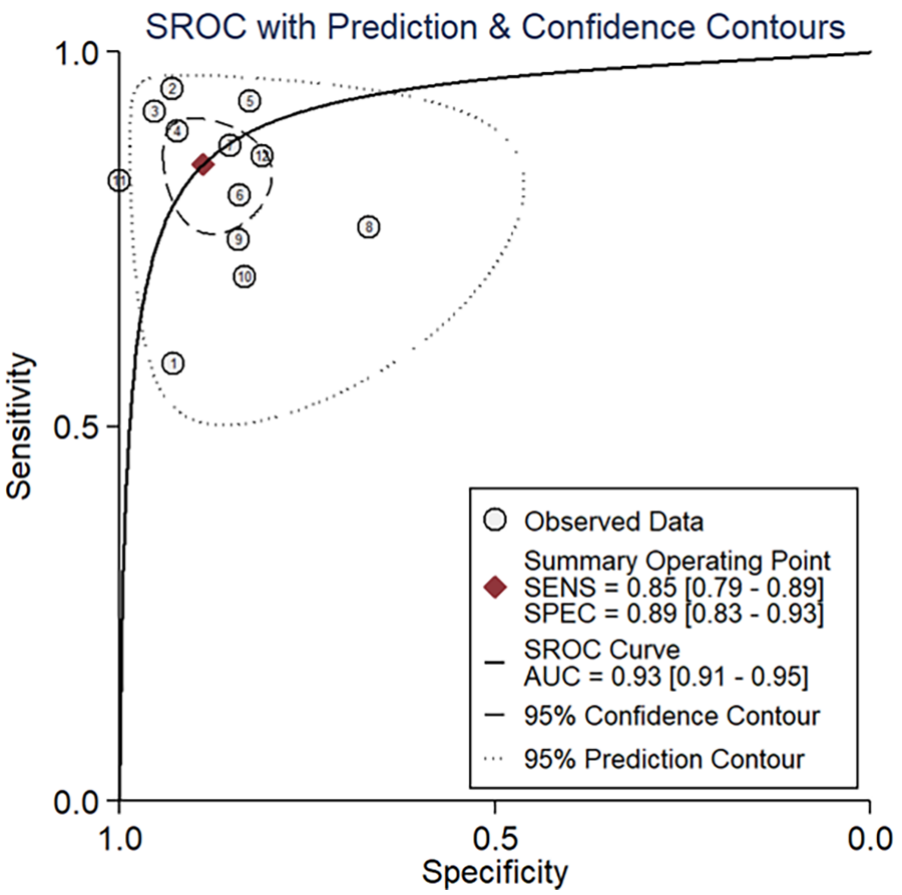
SROC of A-IMR for CMD diagnosis. AUC, area under the curve; SENS, sensitivity; SPEC, specificity; SROC, summary receiver operating characteristic curve.

**Figure 4:**
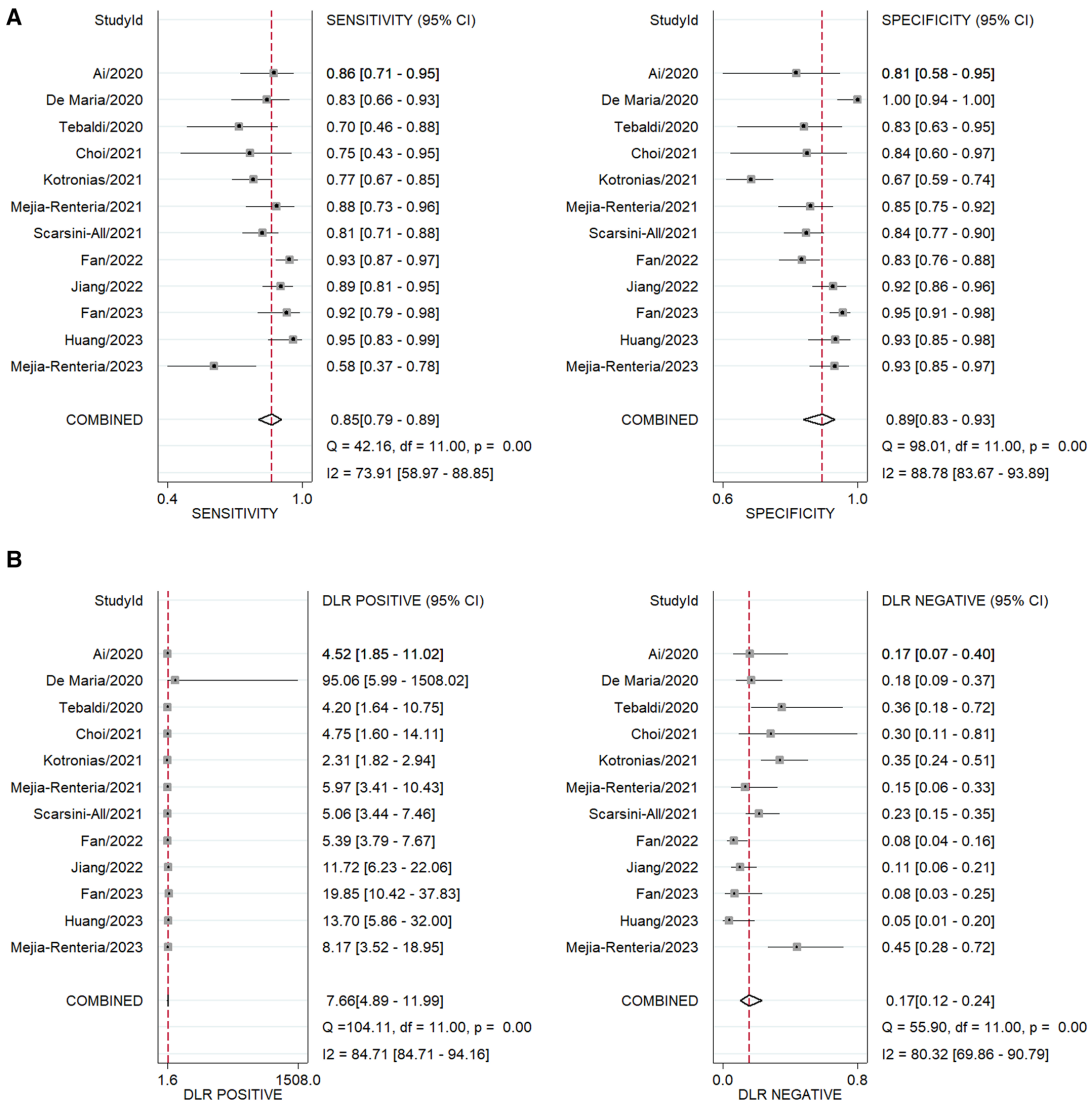
Forest plot of pooled analysis of diagnostic test. (**A**) Sensitivity and specificity. (**B**) +LR and –LR. DLR, diagnostic likelihood ratio.

The area under the sROC was 0.93 (95% CI: 0.91, 0.95) (Figure 3). The pooled sensitivity, specificity, +LR and −LR of A-IMR were 0.85 (95% CI: 0.79, 0.89), 0.89 (95% CI: 0.83, 0.93), 7.66 (95% CI: 4.89, 11.99) and 0.17 (95% CI: 0.12, 0.24), respectively (Figures 4A,B).

We conducted additional subgroup analyses of diagnostic performances using following criteria: within the STEMI population, excluding the STEMI population, excluding retrospective studies, and excluding the INOCA population. The results were consistent irrespective of patient presentation or study type. We also compared the diagnostic performance of different A-IMR systems. The FlashAngio system and QAngio system were used in four and five studies respectively. Both systems exhibited favorable diagnostic accuracy (Supplementary Figure S1). The AccuIMR and AngioPlus systems were used in fewer than three studies, thus precluding meta-analysis.

#### 3.2.3 Heterogeneity analysis

For the threshold effect, the calculated Spearman correlation coefficient was −0.382 (*p*-value = 0.247), which indicated the absence of a threshold effect, as depicted in Supplementary Figure S2A. Regarding the non-threshold effect, with Cochrane-*Q* = 57.40, *p* < 0.001, the *I*^2^-values for pooled sensitivity and specificity stood at 73.9% and 88.8%, respectively, as shown in Figure 3, indicating the exitance of heterogeneity arising from a non-threshold effect. Consequently, we performed a meta-regression to identify the sources of heterogeneity. The meta-regression analysis identified the presence of STEMI as a significant contributor to heterogeneity in both sensitivity and specificity. Additionally, the presence of INOCA and the study design (retrospective or prospective) are also potential sources of heterogeneity in sensitivity (Supplementary Figure S3).

#### 3.2.4 Publication bias

The funnel plot (Supplementary Figure S2B) of showed asymmetry. Deek's test obtained *p*-value of 0.78, indicating that there was no publication bias in statistics.

### 3.3 Prognostic assessment

#### 3.3.1 Prognostic performance

**Figure 5:**
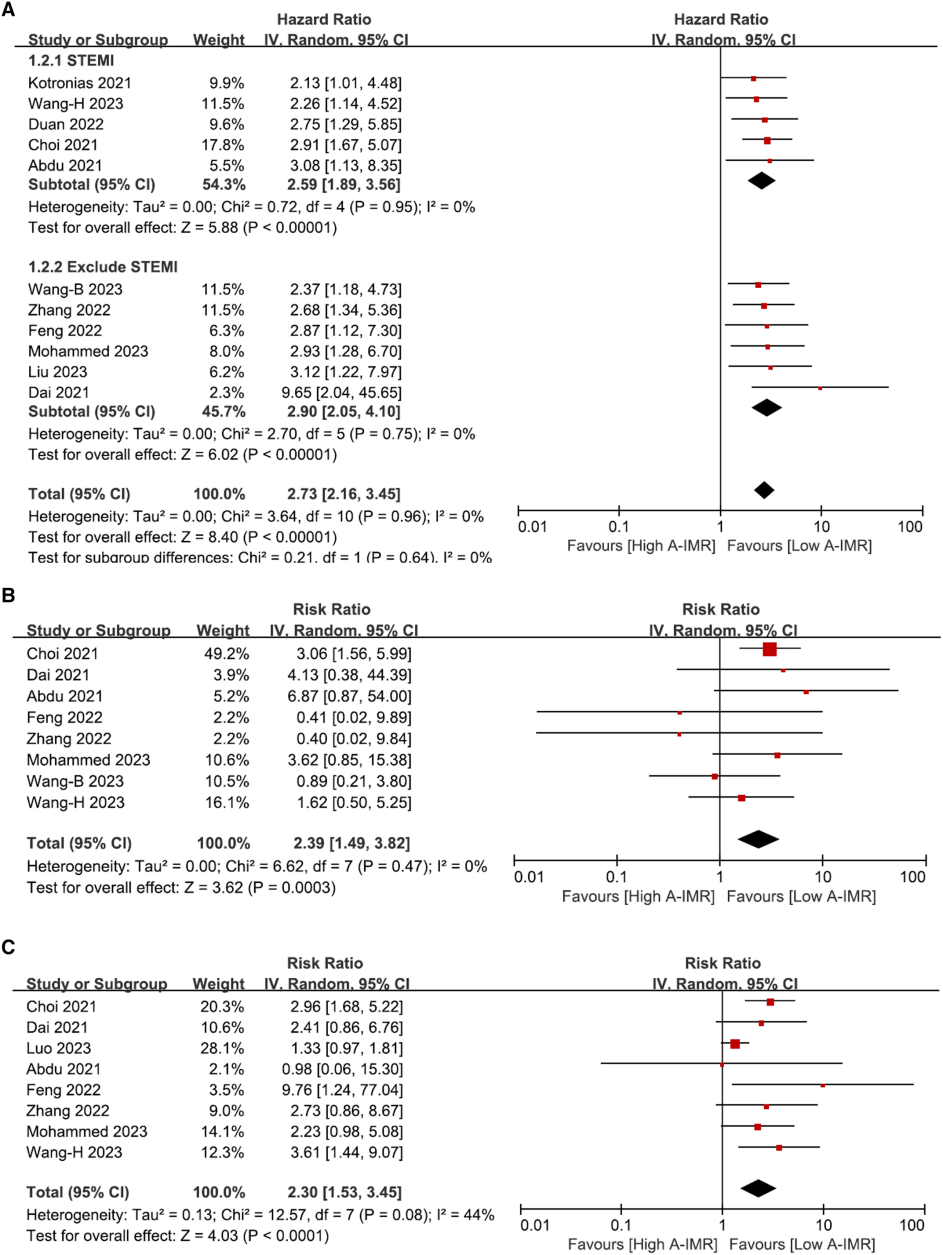
Forest plot of total MACE (**A**), subgroup analysis involving CV death (**B**) and HF readmission (**C**). A-IMR, angiography-derived index of microvascular resistance.

Of the twelve studies in the prognostic meta-analysis, eleven reported the incidence of MACE. Heterogeneity assessment showed an *I*^2^ statistic of 0% with a *p*-value of 0.96, indicating minimal heterogeneity. A random-effects model was employed in the meta-analysis, resulting in a pooled HR of 2.73 (95% CI: 2.16, 3.45). Additionally, we conducted subgroup analyses based on the inclusion of patients with STMEI and the A-FFR value. The findings demonstrated HRs of 2.59 (95% CI: 1.89, 3.56) for the STEMI population and 2.90 (95% CI: 2.05, 4.10) for the population without STEMI. In all studies included where A-FFR ≥ 0.8, HR_MACE_ was 3.10 (2.08, 4.63), similar to the overall cohort. These results suggest a notable increase in the occurrence of long-term MACE in patients diagnosed with CMD by A-IMR, in comparison to those with a normal A-IMR value (Figure 5A).

Eight studies reported the outcome of CV death. Heterogeneity analysis showed an *I*^2^ of 0% and a *p*-value of 0.47. A random-effects model was used for the meta-analysis, yielding a pooled RR of 2.39 (95% CI: 1.49, 3.82) as depicted in Figure 5B. Similarly, eight out of 12 studies reported outcomes of HF hospitalization. Heterogeneity analysis yielded an *I*^2 ^= 44% with a *p*-value of 0.08. We employed random-effects model for the meta-analysis, resulting in a pooled RR of 2.30 with 95% CI: 1.53–3.45 (Figure 5C).

Furthermore, we evaluated outcomes for other independent events, as shown in Supplementary Figure S4, which included all-cause mortality (HR, 1.78, 95% CI: 1.21–2.60), non-fatal AMI (HR, 1.73, 95% CI: 0.92–3.24), and revascularization (HR, 2.12, 95% CI: 1.34–3.35). Due to the limited number of studies addressing outcome of unstable angina hospitalization, we did not perform a pooled statistical analysis.

#### 3.3.2 Quality assessment and sensitivity analysis

NOS scores for the included studies ranged from 5 to 8, indicating the high quality of the literature (Supplementary Table S3). Sensitivity analyses entailed the sequential exclusion of individual studies to determine their influence on the overall effect size. By omitting study Choi (30), the pooled HR of CV death changed to 1.88 (95% CI: 0.97–3.63). The effect size of CMD on MACE, or HF hospitalization remained consistent, even with the exclusion of any individual study, suggesting that the findings are robust (Supplementary Figure S5).

#### 3.3.3 Publication bias

The funnel plot for MACE, CV death and HF hospitalization (Supplementary Figures S6A–C) demonstrates asymmetry, indicating potential publication bias. The Egger's test produced a result of *T*_(MACE) _= 2.75 with a *p*-value of 0.019, indicating a significant publication bias (Supplementary Figure S6D).

Sensitivity analysis involved the stepwise exclusion of individual studies from the analysis to assess their impact on the results. The pooled effects of OAC treatment on all-cause mortality were impacted by the exclusion of 2 studies [Ouellet (16) and Orkaby (8)] and the effect on ischemic stroke was similarly influenced by the exclusion of 2 studies [Orkaby (8) and Subic (7)]. The pooled effects of major bleeding remained unchanged with the omission of a single study.

#### 3.3.4 Publication bias

The funnel plot (Supplementary Figure S1) of OAC treatment on all-cause mortality, major bleeding and ischemic stroke showed asymmetry, indicating possible publication bias. Egger's test obtained t_all−cause mortality_ = −0.749 (*p* = 0.112), t_major bleeding_ = −0.02 (*p* = 0.577), and t_ischemic stroke_ = −0.28 (*p* = 0.260) respectively, indicating that there was no publication bias in statistics (Supplementary Figure S2).

## 4 Discussion

### 4.1 Main findings

In the present study, we reviewed all published articles on diagnostic tests employing the A-IMR technique for CMD diagnosis and prognostic studies predicting adverse CV events. Results indicate that A-IMR provide a favorable diagnostic effect for CMD. CMD, as confirmed by A-IMR, is strongly associated with subsequent adverse CV outcomes. These findings underscore the diagnostic and prognostic value of A-IMR in patients with CMD.

### 4.2 Interpretation

From a mechanistic perspective, CMD predominantly originates from functional changes, such as vasodilator anomalies and microvascular spasms, and structural modifications including adverse arteriole remodeling and intimal thicken (2). Among AMI patients, CMD pathogenesis involves microvascular embolism (42). Within our diagnostic meta-analysis, we stratified the studies/cohorts based on STEMI. We found that irrespective of STEMI presence, the A-IMR consistently demonstrated robust diagnostic efficacy (Figures 5A,B). Some of the included studies induced hyperemia during the A-IMR procedure (13, 25). Theoretically, the induction of hyperemia might counteract microvascular spasm, potentially improving diagnostic accuracy. However, substantial evidence supporting this assertion is still absent. Given the cost and potential risk of hyperemia induction, we recommend against its application in the A-IMR test.

A sequential prognostic meta-analysis emphasized the clinical relevance of A-IMR concerning adverse CV events. The pooled HR for A-IMR in predicting long-term MACE was 2.73 (95% CI: 2.16, 3.45), and consistent in both populations with and without STEMI. Compared with other CMD diagnosis methods, A-IMR did not manifest clear inferiority to T-IMR [HR_MACE_ ranges from 2.2 to 4.1 (43)] or electrocardiography [HR_MACE_ ranges from 1.43 to 1.68 (44)]; contrarily, in comparison to CMR (44, 45) and cardiac PET (46), the HR_MACE_ of A-IMR was comparatively lower.

Nevertheless, the primary advantage of A-IMR is availability. As mentioned above, current methods (including T-IMR, MRI, and cardiac PET) each possess inherent limitations in terms of availability. The benefits of A-IMR are obvious. In contrast to T-IMR, A-IMR avoids the need for additional pressure wires or drugs, presenting a cost-effective solution; it avoids the requirement for intra-coronary operations, emphasizing its simplicity; it avoids the use of adenosine-induced hyperemia, thereby preventing associated hypotension and tachycardia, enhancing its safety profile; it avoids extra data collection as all necessary data can be derived from routine coronary angiography, showcasing its efficiency. In comparison to non-invasive techniques, A-IMR primarily has economic and efficiency advantages. The acquisition and ongoing upkeep of CMR and cardiac PET devices pose significant financial burdens, often hindering their adoption in many healthcare institutions (2); conversely, a A-IMR system can be seamlessly integrated into conventional cathlab. The CMR procedure might extend to an hour and cardiac PET takes up to approximately 40 min, both necessitate substantial patient compliance; A-IMR primarily requires patient cooperation during coronary angiography without further prerequisites. Furthermore, conditions require CMD evaluation encompass INOCA (3) and during the completion of primary percutaneous coronary intervention in AMI cases (47). Each of these contexts can supply the required coronary angiographic imagery for A-IMR. Therefore, A-IMR should be regarded as a non-invasive assessment method.

### 4.3 Clinical application suggestion

In terms of applicability, it is crucial to note that A-IMR demands high-quality coronary angiography images. A-IMR's primary drawback lies in its high demand for coronary angiography images. Additionally, in certain situations such as myocardial bridging, left main artery stenosis, and vascular tortuosity, the utility of A-IMR is limited. However, these scenarios are not uncommon in clinical practice. We found that most of the included studies excluded certain patients during the analysis stage due to insufficient quality of coronary angiography images (Supplementary Figure S4). This proportion is notably high in retrospective studies (up to over 30%). In prospective studies, operators can adjust the angle of projections and quality of coronary angiography. Therefore, we posit that A-IMR is better suited for prospectively assessing patient prognosis rather than relying on retrospective angiographical images for prognostic analysis.

Despite IMR value (primarily based on T-IMR) has been validated for its prognostic value, it does not influence clinical decision-making in most cases. There is currently no evidence supporting the improvement of patient clinical outcomes through drug therapy guided by IMR. Some centers have attempted targeted therapeutic interventions based on IMR, for instance, considering long-term administration of medications like nicorandil and ranolazine to ameliorate microcirculation in patients with IMR > 25 mmHg·s/mm. The availability advantage of A-IMR enables the assessment of CMVD for most patients in clinical settings, thus facilitating evidence-based support for drug therapy guided by IMR.

### 4.4 Comparison with previous studies

Previous investigations have encompassed two meta-analyses that evaluated the diagnostic and prognostic value of A-IMR (48, 49). Our study aligns with the findings of those two analyses. Notably, we have integrated a more expansive and comprehensive evidence in both diagnostic and prognostic dimensions, thereby enhancing the reliability of the results.

We also identified studies employing CMR and CZT-SPECT as reference tests. Within these studies, the AUC of A-IMR for diagnosing CMD were 0.716 (50), 0.821 (51) and 0.84 (7)respectively. In comparison, A-IMR showed superior performance in studies where the T-IMR strategy was used as the reference test. We speculate the main reason is that the A-IMR employs the same rationale as T-IMR (12), resulting in A-IMR measurements that closely align with those of T-IMR.

### 4.5 Strengths & limitations

A key strength of this study is its comprehensive systematic review of A-IMR's diagnostic role in CMD, encompassing a broader range of evidence than earlier research. Additionally, we integrated diagnostic and prognostic meta-analysis, which provided direct evidence of A-IMR in clinical settings.

This study has several limitations. (1) The diagnostic meta-analysis show heterogeneity. We have confirmed that the heterogeneity derived from non-threshold effects and have clarified sources of heterogeneity using meta-regression. (2) In prognostic meta-analysis, Egger's test revealed a publication bias, which might influence the accuracy of the overall results. (3) The prognostic meta-analysis did not incorporate any prospective studies. A-IMR is a newly emerged technology, and prospective studies based on A-IMR might still be ongoing or not yet published. Thus, studies that have been published to date relied on previously documented coronary angiography image for A-IMR calculation. (4) In the prognostic meta-analysis, as depicted in Table 1, the studies employed different definitions of MACE. We based the decision to conduct a pooled MACE analysis on several reasons. Predominantly, only HR of MACE were adjusted via multivariate Cox regression models. Moreover, in the majority of these studies, MACE encompassed HR hospitalization and CV death. These two occurrences were the major contributors to the total MACE count, with other events exerting minimal influence. (5) Considering INOCA is one of the main indications for measuring IMR, a subgroup analysis focusing on INOCA patients should have been conducted. However, only three studies focused on INOCA population. Due to limitations in STATA software, meta-analysis and sROC plotting for outcomes with fewer than four studies were not applicable. Despite these limitations, we maintain that the overall findings of this study remain robust.

## 5 Conclusion

In this systematic review and meta-analysis, we observed that A-IMR demonstrated high diagnostic accuracy for diagnosing CMD in patients with known or suspected CAD. A-IMR also provided robust prognostic value on subsequent MACE or other adverse CV events. Considering the economic and operational benefits associated with A-IMR, we advocate for its broader implementation to curtail healthcare expenses and enhance clinical outcomes for patients.

## Data Availability

The original contributions presented in the study are included in the article/**Supplementary Material**, further inquiries can be directed to the corresponding authors.

## References

[1] Shah SJ Lam CSP Svedlund S Saraste A Hage C Tan RS Prevalence and correlates of coronary microvascular dysfunction in heart failure with preserved ejection fraction: promis-Hfpef. Eur Heart J. (2018) 39(37):3439–50. 10.1093/eurheartj/ehy531 30165580 PMC6927847

[2] Del Buono MG Montone RA Camilli M Carbone S Narula J Lavie CJ Coronary microvascular dysfunction across the spectrum of cardiovascular diseases: JACC state-of-the-art review. J Am Coll Cardiol. (2021) 78(13):1352–71. 10.1016/j.jacc.2021.07.042 34556322 PMC8528638

[3] Knuuti J Wijns W Saraste A Capodanno D Barbato E Funck-Brentano C 2019 esc guidelines for the diagnosis and management of chronic coronary syndromes. Eur Heart J. (2020) 41(3):407–77. 10.1093/eurheartj/ehz425 31504439

[4] Indorkar R Kwong RY Romano S White BE Chia RC Trybula M Global coronary flow reserve measured during stress cardiac magnetic resonance imaging is an independent predictor of adverse cardiovascular events. J Am Coll Cardiol Img. (2019) 12(8 Pt 2):1686–95. 10.1016/j.jcmg.2018.08.018 30409558

[5] El Aidi H Adams A Moons KG Den Ruijter HM Mali WP Doevendans PA Cardiac magnetic resonance imaging findings and the risk of cardiovascular events in patients with recent myocardial infarction or suspected or known coronary artery disease: a systematic review of prognostic studies. J Am Coll Cardiol. (2014) 63(11):1031–45. 10.1016/j.jacc.2013.11.048 24486280

[6] Marinescu MA Löffler AI Ouellette M Smith L Kramer CM Bourque JM. Coronary microvascular dysfunction, microvascular angina, and treatment strategies. JACC Cardiovasc Imaging. (2015) 8(2):210–20. 10.1016/j.jcmg.2014.12.008 25677893 PMC4384521

[7] Dai N Che W Liu L Zhang W Yin G Xu B Diagnostic value of angiography-derived IMR for coronary microcirculation and its prognostic implication after PCI. Front Cardiovasc Med. (2021) 8:735743. 10.3389/fcvm.2021.735743 34722667 PMC8553988

[8] Tsang KH Chan WS Shiu CK Chan MK. The safety and tolerability of adenosine as a pharmacological stressor in stress perfusion cardiac magnetic resonance imaging in the Chinese population. Hong Kong Med J=Xianggang yi xue za zhi. (2015) 21(6):524–7. 10.12809/hkmj144437 26273015

[9] Kunadian V Chieffo A Camici PG Berry C Escaned J Maas A An EAPCI expert consensus document on ischaemia with non-obstructive coronary arteries in collaboration with European society of cardiology working group on coronary pathophysiology & microcirculation endorsed by coronary vasomotor disorders international study group. Eur Heart J. (2020) 41(37):3504–20. 10.1093/eurheartj/ehaa503 32626906 PMC7577516

[10] Everaars H de Waard GA Schumacher SP Zimmermann FM Bom MJ van de Ven PM Continuous thermodilution to assess absolute flow and microvascular resistance: validation in humans using [^15^O]H_2_O positron emission tomography. Eur Heart J. (2019) 40(28):2350–9. 10.1093/eurheartj/ehz245 31327012

[11] Kotecha T Martinez-Naharro A Boldrini M Knight D Hawkins P Kalra S Automated pixel-wise quantitative myocardial perfusion mapping by CMR to detect obstructive coronary artery disease and coronary microvascular dysfunction: validation against invasive coronary physiology. JACC Cardiovasc Imaging. (2019) 12(10):1958–69. 10.1016/j.jcmg.2018.12.022 30772231 PMC8414332

[12] Ai H Feng Y Gong Y Zheng B Jin Q Zhang HP Coronary angiography-derived index of microvascular resistance. Front Physiol. (2020) 11:605356. 10.3389/fphys.2020.605356 33391020 PMC7772433

[13] De Maria GL Scarsini R Shanmuganathan M Kotronias RA Terentes-Printzios D Borlotti A Angiography-derived index of microcirculatory resistance as a novel, pressure-wire-free tool to assess coronary microcirculation in st elevation myocardial infarction. Int J Cardiovasc Imaging. (2020) 36(8):1395–406. 10.1007/s10554-020-01831-7 32409977 PMC7381481

[14] Jiang J Li C Hu Y Li C He J Leng X A novel CFD-based computed index of microcirculatory resistance (IMR) derived from coronary angiography to assess coronary microcirculation. Comput Methods Programs Biomed. (2022) 221:106897. 10.1016/j.cmpb.2022.106897 35636354

[15] Fan Y Fezzi S Sun P Ding N Li X Hu X *In vivo* validation of a novel computational approach to assess microcirculatory resistance based on a single angiographic view. J Pers Med. (2022) 12(11):1978. 10.3390/jpm12111798 36573725 PMC9692562

[16] Wang D Xu X Peng W Pan G. Diagnostic and prognostic value of the angiography-derived index of coronary microvascular resistance in coronary microvascular dysfunction: a systematic review and meta-analysis. PROSPERO. (2023). Available online at: https://www.crd.york.ac.uk/prospero/display_record.php?ID=CRD42023451884

[17] McInnes MDF Moher D Thombs BD McGrath TA Bossuyt PM Clifford T Preferred reporting items for a systematic review and meta-analysis of diagnostic test accuracy studies: the prisma-dta statement. JAMA. (2018) 319(4):388–96. 10.1001/jama.2017.19163 29362800

[18] Page MJ McKenzie JE Bossuyt PM Boutron I Hoffmann TC Mulrow CD The prisma 2020 statement: an updated guideline for reporting systematic reviews. BMJ. (2021) 372:n71. 10.1136/bmj.n71 33782057 PMC8005924

[19] Whiting PF Rutjes AW Westwood ME Mallett S Deeks JJ Reitsma JB Quadas-2: a revised tool for the quality assessment of diagnostic accuracy studies. Ann Intern Med. (2011) 155(8):529–36. 10.7326/0003-4819-155-8-201110180-00009 22007046

[20] Stang A. Critical evaluation of the Newcastle-Ottawa scale for the assessment of the quality of nonrandomized studies in meta-analyses. Eur J Epidemiol. (2010) 25(9):603–5. 10.1007/s10654-010-9491-z 20652370

[21] Zamora J Abraira V Muriel A Khan K Coomarasamy A. Meta-Disc: a software for meta-analysis of test accuracy data. BMC Med Res Methodol. (2006) 6:31. 10.1186/1471-2288-6-31 16836745 PMC1552081

[22] Stata/Se 14.0 for Windows. College Station, TX: StataCorp LLC (2011).

[23] Dwamena B. Midas: Stata Module for Meta-Analytical Integration of Diagnostic Test Accuracy Studies. Ann Arbor, MI: Boston College Department of Economics (2009).

[24] Review Manager (Rev.Man.). 5.4.1 Version ed. Copenhagen, Denmark: The Cochrane Collaboration (2014).

[25] Scarsini R Shanmuganathan M Kotronias RA Terentes-Printzios D Borlotti A Langrish JP Angiography-derived index of microcirculatory resistance [IMR(angio)] as a novel pressure-wire-free tool to assess coronary microvascular dysfunction in acute coronary syndromes and stable coronary artery disease. Int J Cardiovasc Imaging. (2021) 37(6):1801–13. 10.1007/s10554-021-02254-8 33950329

[26] Tebaldi M Biscaglia S Di Girolamo D Erriquez A Penzo C Tumscitz C Angio-based index of microcirculatory resistance for the assessment of the coronary resistance: a proof of concept study. J Interv Cardiol. (2020) 2020:8887369. 10.1155/2020/8887369 33162844 PMC7605930

[27] Mejia-Renteria H Lee JM Choi KH Lee SH Wang L Kakuta T Coronary microcirculation assessment using functional angiography: development of a wire-free method applicable to conventional coronary angiograms. Catheter Cardiovasc Interv. (2021) 98(6):1027–37. 10.1002/ccd.29863 34242489

[28] Huang D Gong Y Fan Y Zheng B Lu Z Li J Coronary angiography-derived index for assessing microcirculatory resistance in patients with non-obstructed vessels: the flash IMR study. Am Heart J. (2023) 263:56–63. 10.1016/j.ahj.2023.03.016 37054908

[29] Fan Y Li C Hu Y Hu X Wang S He J Angiography-based index of microcirculatory resistance (accuimr) for the assessment of microvascular dysfunction in acute coronary syndrome and chronic coronary syndrome. Quant Imaging Med Surg. (2023) 13(6):3556–68. 10.21037/qims-22-961 37284070 PMC10240038

[30] Choi KH Dai N Li Y Kim J Shin D Lee SH Functional coronary angiography-derived index of microcirculatory resistance in patients with st-segment elevation myocardial infarction. JACC Cardiovasc Interv. (2021) 14(15):1670–84. 10.1016/j.jcin.2021.05.027 34353599

[31] Kotronias RA Terentes-Printzios D Shanmuganathan M Marin F Scarsini R Bradley-Watson J Long-term clinical outcomes in patients with an acute st-segment-elevation myocardial infarction stratified by angiography-derived index of microcirculatory resistance. Front Cardiovasc Med. (2021) 8:717114. 10.3389/fcvm.2021.717114 34557531 PMC8452918

[32] Mejia-Renteria H Wang L Chipayo-Gonzales D van de Hoef TP Travieso A Espejo C Angiography-derived assessment of coronary microcirculatory resistance in patients with suspected myocardial ischaemia and non-obstructive coronary arteries. EuroIntervention. (2023) 18(16):E1348-+. 10.4244/eij-d-22-00579 36534493 PMC10068857

[33] Wang H Wu Q Yang L Chen L Liu WZ Guo J Application of AMR in evaluating microvascular dysfunction after ST-elevation myocardial infarction. Clin Cardiol. (2024) 47(2):e24196. 10.1002/clc.24196 37997762 PMC10823552

[34] Abdu FA Liu L Mohammed AQ Yin G Xu B Zhang W Prognostic impact of coronary microvascular dysfunction in patients with myocardial infarction with non-obstructive coronary arteries. Eur J Intern Med. (2021) 92:79–85. 10.1016/j.ejim.2021.05.027 34092485

[35] Liu L Dai N Yin G Zhang W Mohammed AQ Xu S Prognostic value of combined coronary angiography-derived IMR and myocardial perfusion imaging by CZT SPECT in INOCA. J Nucl Cardiol. (2023) 30(2):684–701. 10.1007/s12350-022-03038-w 35918592

[36] Duan Y Wang YW Zhang M Li Z Chen L Miao H Computational pressure-fluid dynamics applied to index of microcirculatory resistance, predicting the prognosis of drug-coated balloons compared with drug-eluting stents in sTEMI patients. Front Physiol. (2022) 13:16. 10.3389/fphys.2022.898659 PMC9171027 35685283

[37] Mohammed AQ Abdu FA Su Y Liu L Yin G Feng Y Prognostic significance of coronary microvascular dysfunction in patients with heart failure with preserved ejection fraction. Can J Cardiol. (2023) 39(7):971–80. 10.1016/j.cjca.2023.04.011 37086837

[38] Zhang W Singh S Liu L Mohammed AQ Yin G Xu S Prognostic value of coronary microvascular dysfunction assessed by coronary angiography-derived index of microcirculatory resistance in diabetic patients with chronic coronary syndrome. Cardiovasc Diabetol. (2022) 21(1):222. 10.1186/s12933-022-01653-y 36309724 PMC9618191

[39] Feng C Abdu FA Mohammed AQ Zhang W Liu L Yin G Prognostic impact of coronary microvascular dysfunction assessed by caIMR in overweight with chronic coronary syndrome patients. Front Endocrinol. (2022) 13:922264. 10.3389/fendo.2022.922264 PMC9399838 36034462

[40] Luo D Wu H Zhou WJ Zhang J Jin X Xu CW Angio-based coronary functional assessment predicts 30-day new-onset heart failure after acute myocardial infarction. ESC Heart Failure. (2023) 13:2914–926. 10.1002/ehf2.14452 PMC10567646 37455355

[41] Wang B Gao Y Zhao Y Jia P Han J Li H Prognostic value of angiography-derived index of microcirculatory resistance in patients with coronary artery disease undergoing rotational atherectomy. Rev Cardiovasc Med. (2023) 24(5):131. 10.31083/j.rcm2405131 PMC11273008 39076748

[42] Kleinbongard P Heusch G. A fresh look at coronary microembolization. Nat Rev Cardiol. (2022) 19(4):265–80. 10.1038/s41569-021-00632-2 34785770 PMC8593642

[43] Canu M Khouri C Marliere S Vautrin E Piliero N Ormezzano O Prognostic significance of severe coronary microvascular dysfunction post-PCI in patients with STEMI: a systematic review and meta-analysis. PLoS One. (2022) 17(5):e0268330. 10.1371/journal.pone.0268330 35576227 PMC9109915

[44] Yang Y Li W Zhu H Pan XF Hu Y Arnott C Prognosis of unrecognised myocardial infarction determined by electrocardiography or cardiac magnetic resonance imaging: systematic review and meta-analysis. BMJ. (2020) 369:m1184. 10.1136/bmj.m1184 32381490 PMC7203874

[45] Ricci F Khanji MY Bisaccia G Cipriani A Di Cesare A Ceriello L Diagnostic and prognostic value of stress cardiovascular magnetic resonance imaging in patients with known or suspected coronary artery disease: a systematic review and meta-analysis. JAMA Cardiol. (2023) 8(7):662–73. 10.1001/jamacardio.2023.1290 37285143 PMC10248816

[46] Gebhard C Fiechter M Herzog BA Lohmann C Bengs S Treyer V Sex differences in the long-term prognostic value of (13)N-ammonia myocardial perfusion positron emission tomography. Eur J Nucl Med Mol Imaging. (2018) 45(11):1964–74. 10.1007/s00259-018-4046-8 29779046

[47] Fahrni G Wolfrum M De Maria GL Cuculi F Dawkins S Alkhalil M Index of microcirculatory resistance at the time of primary percutaneous coronary intervention predicts early cardiac complications: insights from the oxami (Oxford study in acute myocardial infarction) cohort. J Am Heart Assoc. (2017) 6(11):14. 10.1161/jaha.116.005409 PMC5721736 29113999

[48] Fernández-Peregrina E Garcia-Garcia HM Sans-Rosello J Sanz-Sanchez J Kotronias R Scarsini R Angiography-derived versus invasively-determined index of microcirculatory resistance in the assessment of coronary microcirculation: a systematic review and meta-analysis. Catheter Cardiovasc Interv. (2022) 99(7):2018–25. 10.1002/ccd.30174 35366386

[49] Li W Takahashi T Rios SA Latib A Lee JM Fearon WF Diagnostic performance and prognostic impact of coronary angiography-based index of microcirculatory resistance assessment: a systematic review and meta-analysis. Catheter Cardiovasc Interv. (2022) 99(2):286–92. 10.1002/ccd.30076 35019220

[50] Sheng XC Qiao ZQ Ge H Sun JT He J Li Z Novel application of quantitative flow ratio for predicting microvascular dysfunction after st-segment-elevation myocardial infarction. Catheter Cardiovasc Interv. (2020) 95:624–32. 10.1002/ccd.28718 31912991

[51] Shin D Kim J Choi KH Dai N Li Y Lee SH Functional angiography-derived index of microcirculatory resistance validated with microvascular obstruction in cardiac magnetic resonance after STEMI. Revista Espanola de Cardiologia. (2022) 75(10):786–96. 10.1016/j.rec.2022.01.004 35249841

