# Supplementary material for: Diagnostic and prognostic value of angiography-derived index of microvascular resistance: a systematic review and meta-analysis
Source: Front Cardiovasc Med. 2024 Apr 15;11:1360648. doi: 10.3389/fcvm.2024.1360648 (PMC11057370; doi:10.3389/fcvm.2024.1360648)
Supplement: Supplementary file 1 [file Datasheet1.docx]

Supplementary Material

Clinical Benefits of Oral Anticoagulants in Atrial Fibrillation Patients with Dementia: A Systematic Review and Meta-Analysis

**Dayang Wang1,2†, Xiaoming Li1†, Wei Feng1, Hufang Zhou1,2, Wenhua Peng2*, Xian Wang2***

*** Correspondence:** Xian Wang. wx650515@126.com.

Wenhua Peng. pengwenhua76@163.com.

# Supplementary Figures and Tables

For more information on Supplementary Material and for details on the different file types accepted, please see [here](https://www.frontiersin.org/guidelines/author-guidelines#supplementary-material).

## Supplementary Figures


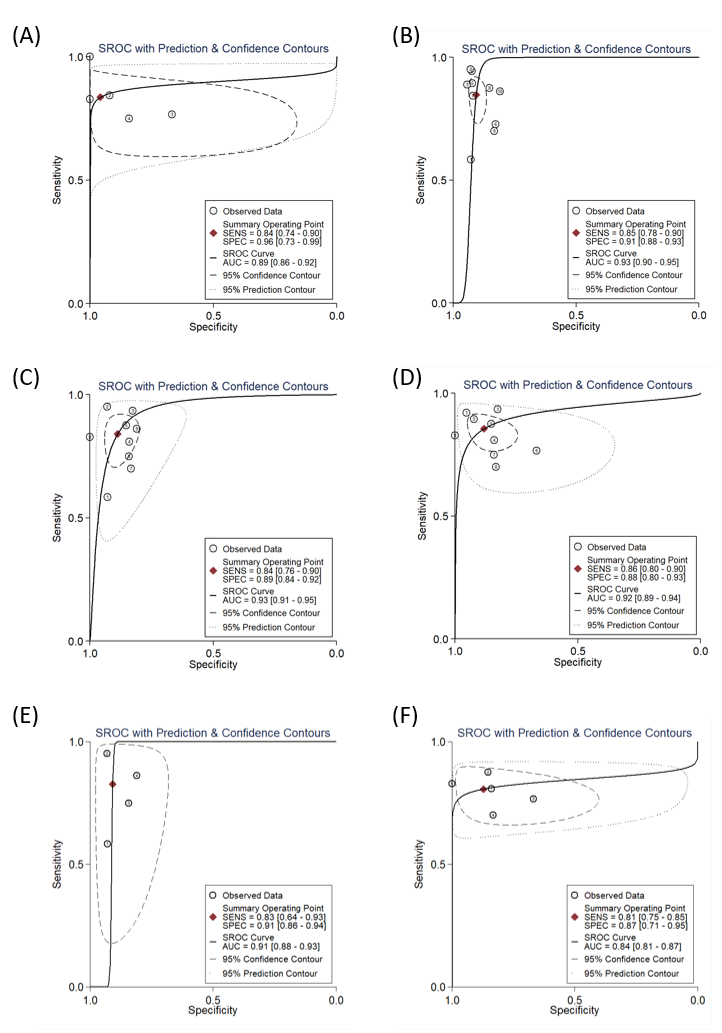


**Supplementary Figure S1.** Subgroup analysis results displayed by the sROC, sensitivity and specificity of STEMI population(A) , excluding the STEMI population (B）, excluding retrospective studies (C), and excluding the INOCA population (D). We also conducted sub-group analysis across different A-IMR systems : FlashAngio (E) and QAngio (F) . Abbreviations: INOCA, ischemia with non-obstructive coronary arteries. SROC, summary receiver operating characteristic curve. STEMI, ST elevated myocardial infarction.


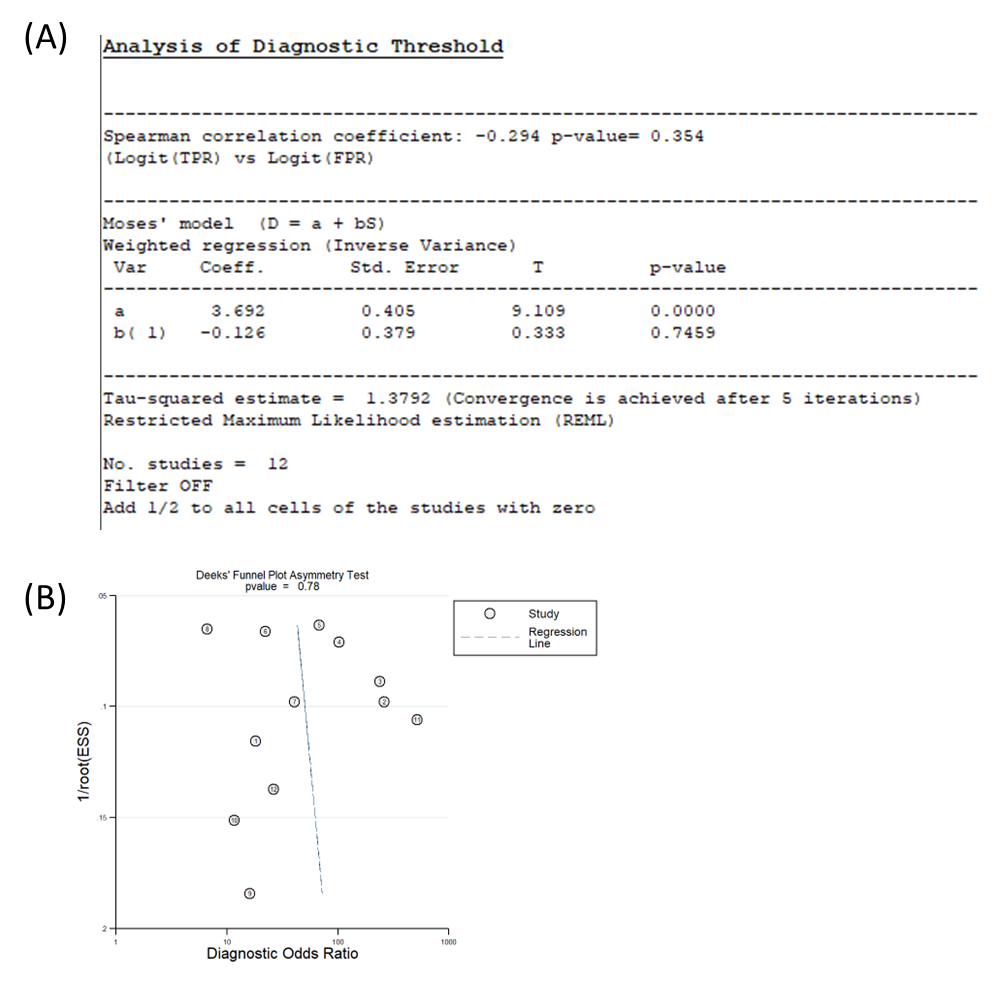
**Supplementary Figure S2.** The meta-regression analysis of diagnostic meta-analysis(A), and diagnostic odds ratio and diagnostic score (B).


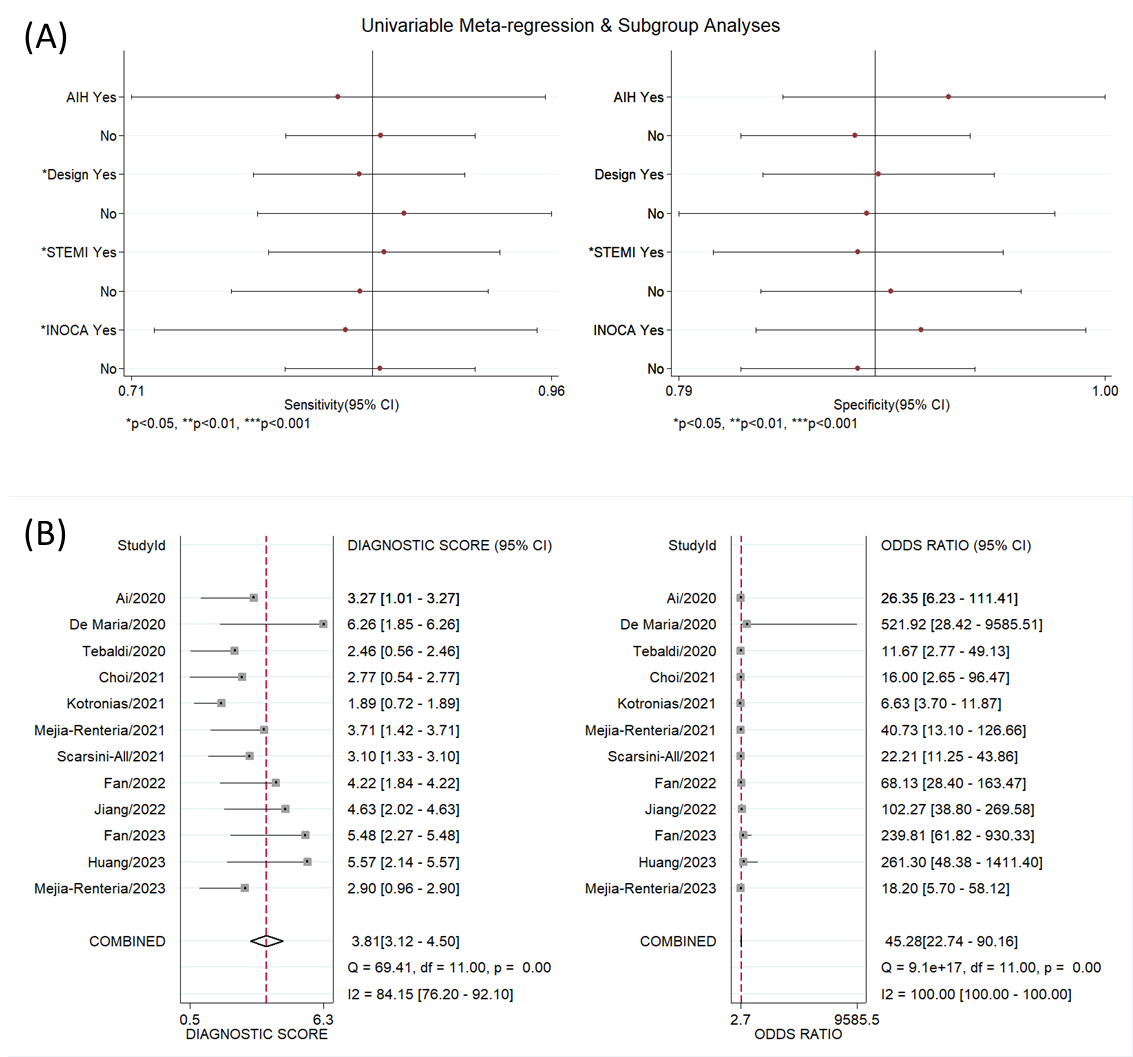


**Supplementary Figure S3.** The meta-regression analysis of diagnostic meta-analysis(A), and diagnostic odds ratio and diagnostic score (B).


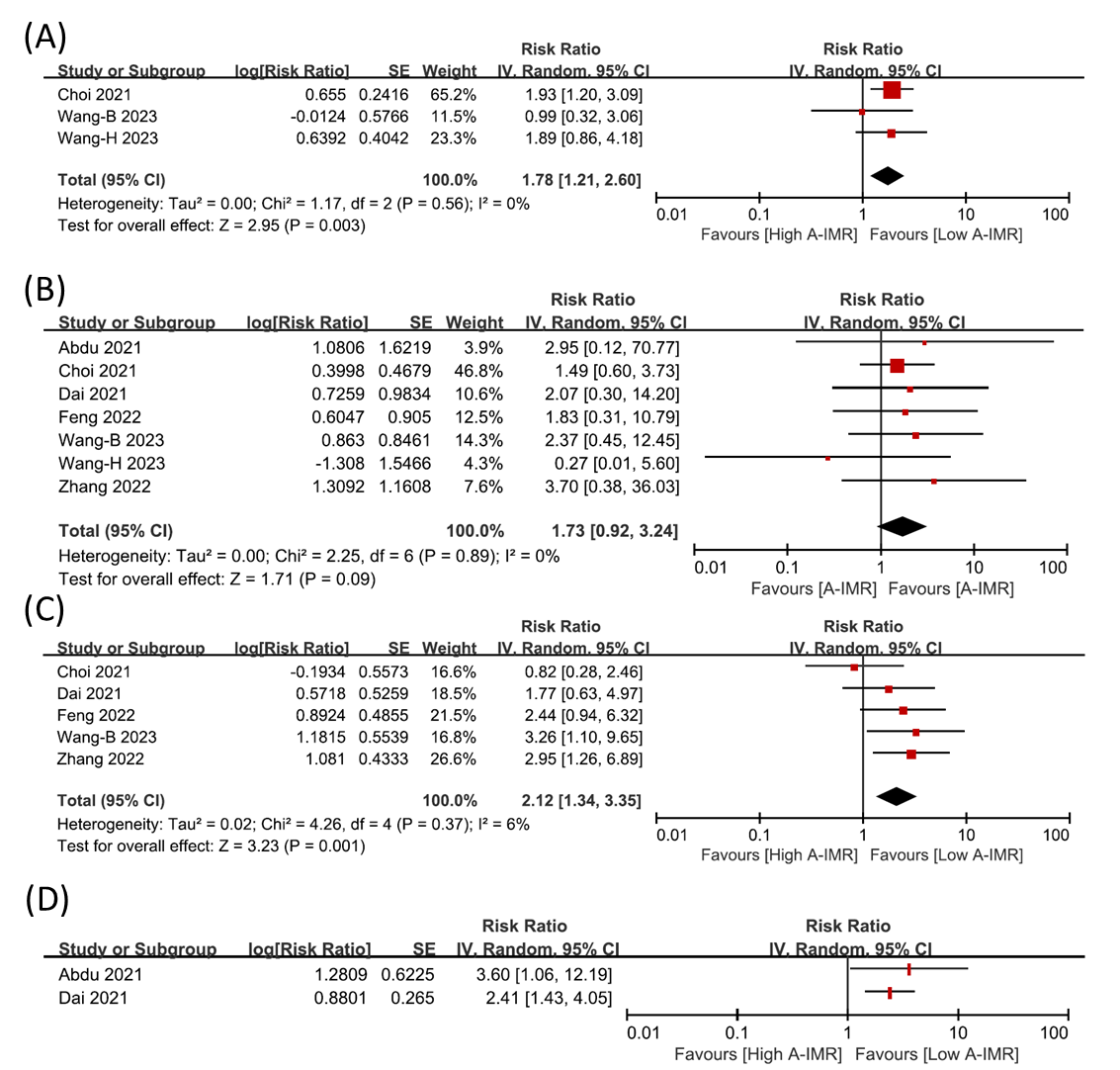


**Supplementary Figure S4.** Forest plot of (A)all-cause mortality, (B)Myocardial Infarction, (C) Revascularization, (D)UA hospitalization.


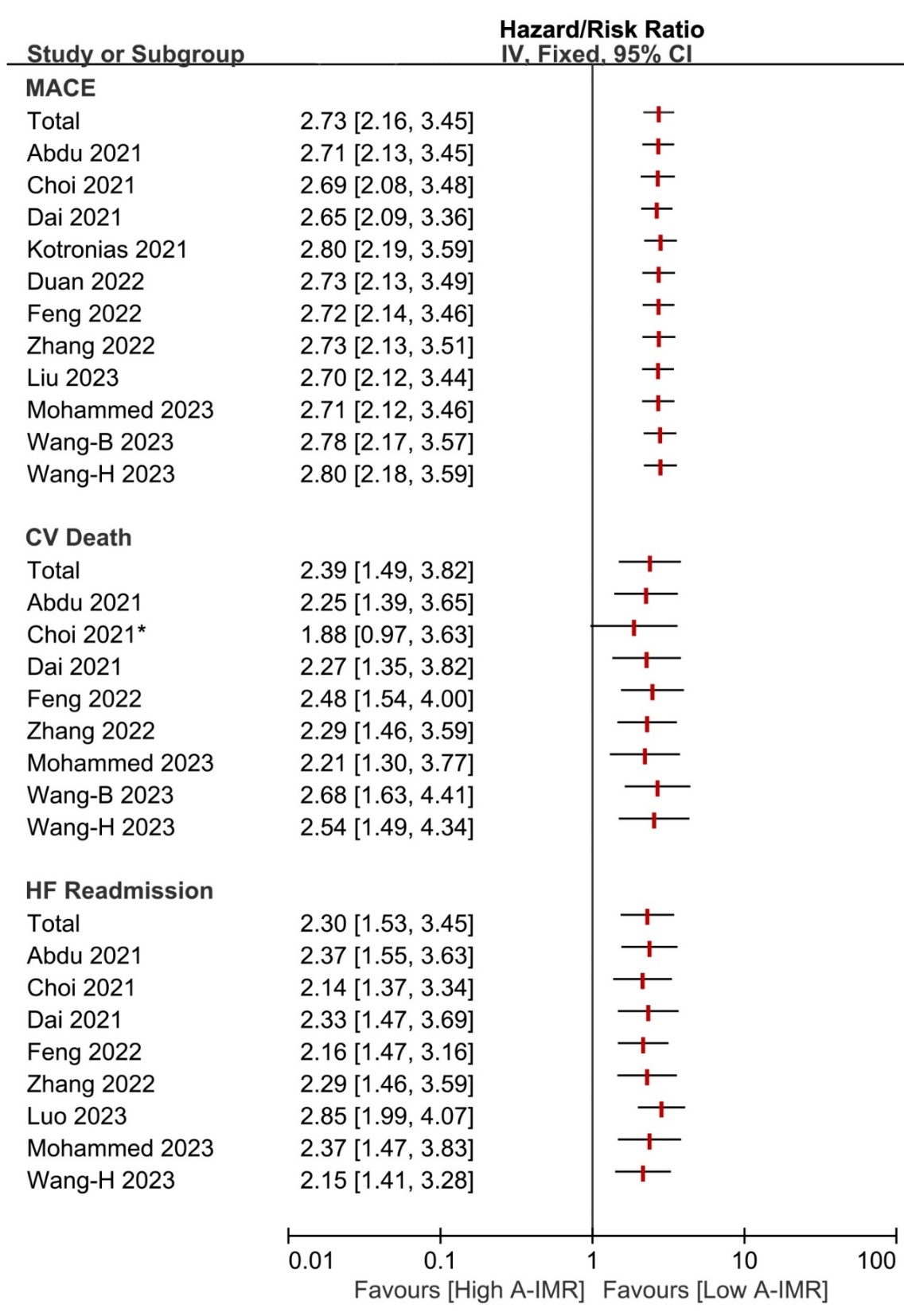


**Supplementary** **Figure S5.** Sensitive analysis of included studies. *Omitting this study may impact the pooled results of the meta-analysis. Abbreviations: CI: confidence interval.


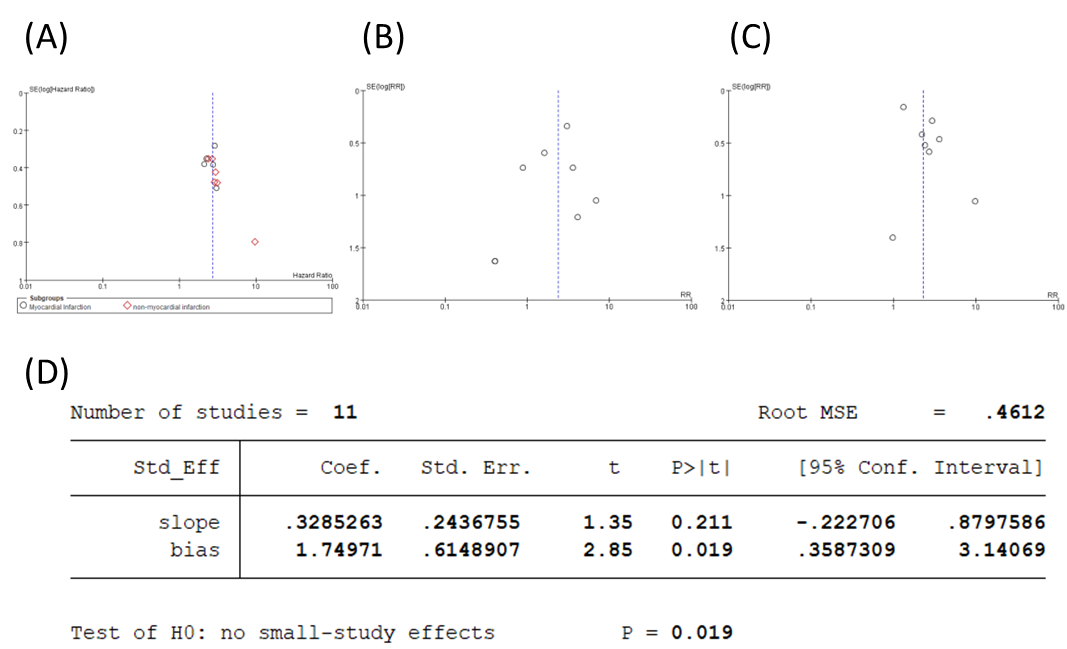


**Supplementary** **Figure S6.** Publication bias assessment of prognostic meta-analysis. Funnel Plot of MACE (A), CV death (B) and HF hospitalization(C). The assessment of publication bias of MACE were conduct additionally using Egger’s test (D).

## Supplementary Tables

**Supplementary Table S1.** Search Strategy

| Database | Search Strategy |
| --- | --- |
| PubMed | ((index of microvascular resistance [Title/Abstract]) OR (index of microcirculatory resistance [Title/Abstract]) OR (coronary microvascular dysfunction [Title/Abstract]) OR (coronary microcirculatory dysfunction [Title/Abstract])) AND ((angiography-derived [Title/Abstract]) OR (non-invasive [Title/Abstract]) OR (angiography derived [Title/Abstract]) OR (angiography based [Title/Abstract]) OR (angio-based [Title/Abstract]) OR (pressure wire free [Title/Abstract]) OR (Ca-IMR [Title/Abstract]) OR (QAngio [Title/Abstract]) OR (AccuIMR [Title/Abstract]) OR (IMRAngio [Title/Abstract])) |
| Embase | ((index of microvascular resistance):ti,ab,kw OR (index of microcirculatory resistance):ti,ab,kw OR (coronary microvascular dysfunction):ti,ab,kw OR (coronary microcirculatory dysfunction):ti,ab,kw) AND ((angiography-derived):ti,ab,kw OR (non-invasive):ti,ab,kw OR (angiography derived):ti,ab,kw OR (angiography based):ti,ab,kw OR (angio-based):ti,ab,kw OR (pressure wire free):ti,ab,kw OR (Ca-IMR):ti,ab,kw OR (QAngio):ti,ab,kw OR (AccuIMR):ti,ab,kw OR (IMRAngio):ti,ab,kw) |
| Cochrane Library | ((index of microvascular resistance):ti,ab,kw OR (index of microcirculatory resistance):ti,ab,kw OR (coronary microvascular dysfunction):ti,ab,kw OR (coronary microcirculatory dysfunction):ti,ab,kw) AND ((angiography-derived):ti,ab,kw OR (non-invasive):ti,ab,kw OR (angiography derived):ti,ab,kw OR (angiography based):ti,ab,kw OR (angio-based):ti,ab,kw OR (pressure wire free):ti,ab,kw OR (Ca-IMR):ti,ab,kw OR (QAngio):ti,ab,kw OR (AccuIMR):ti,ab,kw OR (IMRAngio):ti,ab,kw) |
| Web of Science | #1 TS=(index of microvascular resistance OR (index of microcirculatory resistance OR coronary microvascular dysfunction OR coronary microcirculatory dysfunction)  #2 TS=(angiography-derived OR non-invasive OR angiography derived OR angiography based OR angio-based OR pressure wire free OR Ca-IMR OR QAngio OR AccuIMR OR IMRAngio)  #3 #1 AND #2 |

**Supplementary Table S2**. Adjusted confounders of multivariate analysis in studies included in prognostic prediction analysis

| Table S2. Adjusted confounders of multivariate analysis in studies included in prognostic prediction analysis. | |
| --- | --- |
| Study ID | Adjusted confounders |
| Abdu 2021 | gender, age, traditional cardiovascular risk factors (BMI, smoking history, diabetes, hypertension, hyperlipidemia, atrial fibrillation, and heart failure), SBP, DBP, HR, LVEF, ECG findings, angiographic characteristics, biochemical parameters (TC, TG, HDL-C, LDL-C, cTnT, CK-MB, myoglobin, and NT-proBNP), medications (statins, aspirin, ACEI/ARB, β-BLOCK, and CCB) and caIMR |
| Choi 2021 | age, sex, left ventricular ejection fraction, and post-PCI angio-FFR values |
| Dai 2021 | age, sex, left ventricular ejection fraction, and post-PCI angio-FFR values |
| Kotronias 2021 | clinical, procedural, angiographic and echocardiographic variables |
| Duan 2022 | Age, sex, Systolic BP, Heart rate, BMI, hypertension, Diabetes, smoking, DtoB, KILLIP level, LVEF, hsTNI, CK-MB, CRP, Cr, LDL-C, Aspirin, P2Y12 inhibitors, Statins, Beta-blocker, RAASI, diuretics |
| Feng 2022 | cardiac risk variables and parameters |
| Zhang 2022 | caIMR, sex, BMI, Hyperlipidemia, Hypertension, LVEF, Age, Smoking, Atrial fibrillation, CAD, CKD, PCI performed, FBG, HbA1c, TC, LDL-C, Cr, eGFR, Aspirin, P2Y12 receptor antagonist, Statin, ACEI/ARB, Beta blocker, CCB |
| Liu 2023 | age, gender, body mass index (BMI), smoking history, diabetes, hypertension, hyperlipidemia, echocardiographic, LVEF, NT-proBNP, CMD, abnormal MPI, and stress total perfusion defects (TPD) |
| Luo 2023 | NOT Applicable* |
| Mohammed 2023 | age, sex, BMI, smoking, hypertension, diabetes, chronic kidney disease, atrial fibrillation, laboratory parameters, echocardiography values, along with caIMR. |
| Wang-B 2023 | sex, age, hypertension, diabetes mellitus, hyperlipidemia, smoking, previous PCI, diameter of stenosis, left main stem lesion, triple vessel lesion, angle of calcification, MLA, plaque load, single spin duration, and number |
| Wang-H 2023 | age, male, body mass index, hypertension, diabetes, hyperlipidemia, Previous stroke, HBA1C, BNP, albumin, peak troponin I, random blood glucose, LVEF, and AMR ≥250mmHg*s/m. |
| *Study Luo 2023 was not included in the pooled analysis of MACE; adjusted confounders were not applicable. | |

**Supplementary Table S3**. NOS score of studies included in prognostic prediction analysis.

| Table S3. NOS score of studies included in prognostic prediction analysis. | | | | | | | | | |
| --- | --- | --- | --- | --- | --- | --- | --- | --- | --- |
| Study ID | Selection | | | | Comparability | Outcomes | | | Total |
|  | Representativeness of the exposed cohort | Selection of the non-exposed cohort | Ascertainment of exposure | Demonstration that outcome of interest was not present at start of study |  | Assessment of outcome | Was follow-up long enough for outcomes to occur | Adequacy of follow up of cohorts |  |
| Abdu 2021 | * | * | * | - | ** | * | * | * | 8 |
| Choi 2021 | * | * | * | - | ** | * | * | - | 7 |
| Dai 2021 | * | * | * | - | * | * | * | - | 6 |
| Kotronias 2021 | * | * | * | - | * | * | * | * | 7 |
| Duan 2022 | * | - | * | - | * | * | * | * | 6 |
| Feng 2022 | * | * | * | - | * | * | * | - | 6 |
| Zhang 2022 | * | * | * | - | * | * | * | - | 6 |
| Liu 2023 | - | * | * | - | * | * | * | - | 5 |
| Luo 2023 | * | * | * | - | - | * | - | * | 5 |
| Mohammed 2023 | * | * | * | - | ** | * | * | - | 7 |
| Wang-B 2023 | * | * | * | - | * | * | * | * | 7 |
| Wang-H 2023 | * | * | * | - | - | * | * | * | 6 |
|  | | | | | | | | |  |

**Supplementary Table S4**. Number of cases excluded in analysis stage and reasons.

| Table S4. Number of cases excluded in analysis stage and reasons. | | |
| --- | --- | --- |
| Study ID | Number of Cases | Reasons for Exclusion |
| Ai2020 | - | Not mentioned. |
| DeMaria2020 | 2/47 | Poor angiogram quality for QFR Measurement. |
| Tebaldi2020 | - | Not mentioned. |
| Abdu2021 | 45/154 | Poor angiogram quality for ca-IMR measurement. |
| Choi 2021 | 3/34 | On the basis of image quality of coronary angiography. |
| Dai | 7/187 | No optimal projections for reconstruction |
| Kotronias2021 | 106/368 | Missing record of mean aortic pressure; irretrievable angiograms; unsuitable projections for 3D reconstruction; technically unfeasible (vessel overlap, foreshortening, poor opacification). |
| Mejia2021 | 9/139 | Two complementary optimal x-ray views not available; poor image quality. |
| Scarsini2021 | - | Not mentioned. |
| Duan2022 | 18/213 | Poor image quality. |
| Fan2022 | 46/303 | Poor angiographic quality for AMR computation. |
| Feng2022 | 82/372 | Poor contrast opacification; serious vascular overlap or distortion of the target vessel; poor angiographic image quality are insufficient to demonstrate the contour detection requested by the FLASH software |
| Jiang2022 | 21/227 | Poor image quality; Insufficient injected contrast; Only one projection;  Projections not >25° apart (n=5); Excessive overlap of vessels (n=6) |
| Fan2023 | 82/245 | Unanalyzable poor image quality (n=7); Poor contrast opacification (n=4); Only one projection (n=51); Projections not ≥25° apart (n=9); Severe overlap or distortion of target vessels (n=11) |
| Huang2023 |  | Not mentioned. |
| Liu 2023 | 21/172 | for unsuccessful caIMR measurement due to inadequate image quality of the coronary angiograms |
| Luo2023 | 233/1243 | 160 poor angiographic image quality73 severe vessel tortuosity or overlap |
| Mejia2023 | 11/115 | Low image quality; no complementary, valid views for QFR; calibration failure; non-optimal intracoronary pressure-flow traces; pressure drift not admitted by the core lab; ventricularised curves. |
| Mohammed2023 | 16/238 | Inadequate quality for CAG. |
| WangB2023 | 5/123 | images did not meet the requirements |
| WangH2023 | 5/514 | Poor quality of angiographic images; presence of severe vascular curvature, and overlap. |
|  |  |  |
